# Evaluación de la implementación del protocolo de manejo de coinfección de tuberculosis y VIH en los servicios de asistencia especializada del estado de Ceará, Brasil

**DOI:** 10.26633/RPSP.2017.48

**Published:** 2017-04-21

**Authors:** Silvia Helena Bastos de Paula, Telma Alves Martins, Sheila Maria Santiago Borges, Christiana Maria de Oliveira Nogueira, Valderina Freire Ramos

**Affiliations:** 1 Instituto de Saúde, Secretaria de Estado da Saúde de São Paulo La correspondencia se debe dirigir a Silvia Helena Bastos de Paula São Paulo Brasil Instituto de Saúde, Secretaria de Estado da Saúde de São Paulo, São Paulo, Brasil. La correspondencia se debe dirigir a Silvia Helena Bastos de Paula.; 2 Área técnica de DST HIV/aids Secretaria da Saúde do Estado do Ceará Ceará Brasil Área técnica de DST HIV/aids, Secretaria da Saúde do Estado do Ceará, Ceará, Brasil.; 3 Área técnica de tuberculose Secretaria da Saúde do Estado do Ceará Ceará Brasil Área técnica de DST HIV/aids, Secretaria da Saúde do Estado do Ceará, Ceará, Brasil.

**Keywords:** Tuberculosis, VIH, servicios de salud, protocolos, Brasil, Tuberculosis, HIV, protocols, health services, Brazil

## Abstract

**Objetivos.:**

Identificar barreras y estrategias para la implementación del protocolo “Manejo de coinfección por tuberculosis y virus de inmunodeficiencia humana (TBC/VIH)” en los servicios de asistencia especializada (SAE) de Ceará, mediante investigación evaluativa.

**Métodos.:**

Estudio desarrollado siguiendo la metodología iPIER, una nueva iniciativa con el propósito de apoyar mejoras en la ejecución de programas de salud a través de investigaciones integradas en ellos acerca de su ejecución. Se recogieron datos de estructura y procesos de 22 servicios y se exploraron las barreras mediante cuatro grupos focales, con 28 participantes, desde el punto de vista del equipo de salud, los administradores y los usuarios. Las discusiones fueron transcritas e interpretadas según los objetivos del estudio.

**Resultados.:**

Los datos de estructura y procesos revelaron que seis servicios realizan acciones de manejo de coinfección TB/VIH y 16, no lo hacen. Las barreras fueron: desconocimiento del protocolo en los equipos, ausencia de guías de práctica clínica en los servicios, inserción de los SAE en los tres niveles del sistema de salud, espacios inadecuados para tratar enfermedades de transmisión aérea y falta de comunicación con los sectores de atención primaria de salud para los traslados. Se discutieron los resultados con equipos y administradores en seminarios en los servicios y con los responsables de los programas de enfermedades de transmisión sexual, virus de inmunodeficiencia humana, sida, hepatitis y tuberculosis.

**Conclusiones.:**

El diálogo directo entre administradores, ejecutores, usuarios e investigadores generó conocimiento sobre los servicios y elaboración conjunta de modificaciones de flujos para la aceptación y utilización del protocolo; sin embargo, entre los usuarios persiste la resistencia para adherirse al tratamiento.

## Abstract (en)

***Objectives*.:**

Identify barriers and strategies for implementation of the protocol for managing tuberculosis/human immunodeficiency virus (TB/HIV) coinfection in specialized care services in Ceará state, through evaluation research.

***Methods*.:**

The study followed iPIER methodology, a new initiative to help improve the implementation of health programs through the use of research that is conducted as a part of the implementation process. Data were collected on the structure and processes of 22 services and barriers were explored in four focus groups with 28 participants, from the standpoint of the health team, administrators, and users. The discussions were transcribed and interpreted with regard to the objectives of the study.

***Results*.:**

The information on structure and process revealed that six services manage TB/HIV coinfection and 16 do not. The barriers were: team members were unaware of the protocol; lack of clinical practice guidelines in the services; specialized care services working at all three levels of the health system; inadequate spaces for treatment of airborne diseases; and lack of communication with primary health care sectors for patient transfers. The results were discussed with teams and administrators in seminars held in the services and with program managers for sexually transmitted infections, human immunodeficiency virus, AIDS, hepatitis, and tuberculosis.

***Conclusions*.:**

Direct dialogue between administrators, implementers, users, and researchers generated knowledge about the services and led to joint preparation of modifications in workflow aimed at acceptance and use of the protocol; however, users continue to resist adherence to treatment.

La tuberculosis (TB) está relacionada con las malas condiciones de vida y es un agravante para personas con infección por virus de inmunodeficiencia humana (VIH), con repercusión en la mortalidad por sida en Brasil (1), siendo una realidad para toda América Latina (2). Las personas con esta coinfección son hasta 34 veces más propensas a desarrollar tuberculosis en comparación con la población general y, con frecuencia, el diagnóstico de infección por VIH ocurre durante el desarrollo de la tuberculosis (3).

La red pública para el tratamiento de TB en Brasil fue estructurada y descentralizada desde los años noventa con políticas de control basadas en la atención primaria de la salud. Por otro lado, la red de asistencia de sida es relativamente reciente y su control se concentra en los niveles de atención secundario y terciario (4). En el estado de Ceará, la pobreza y la baja escolaridad también contribuyen en el aumento de la relevancia de la coinfección TB/VIH, aumento de reactivación de infecciones latentes y problemas de adhesión al tratamiento, hubo una disminución en el porcentaje de cura de 73,2% en 2004 a 59,2% en 2014, y la tasa de abandono de 12,7% en 2014 está por encima de lo aceptable por la Organización Mundial de la Salud (OMS), que es de 5% (5–8).

En Brasil, la coinfección por TB/VIH es objeto de una política del Ministerio de Salud, que indica la realización de pruebas de VIH para todos los afectados por TB, y prueba tuberculínica (PPD) y tratamiento de TB pulmonar activa e infección latente por *M. tuberculosis* (ILTB) en personas infectadas con VIH (9).

En 2013, se adoptó el “Protocolo clínico para manejo de la coinfección TB/VIH” que forma parte del Protocolo clínico de directrices terapéuticas para el manejo de la infección por VIH en adultos (10), para su implementación en los servicios de atención especializada (SAE) a personas con infección por VIH. Aún después de la adopción del nuevo protocolo por el Ministerio de Salud, aún hoy persiste un desfase significativo entre la media nacional y lo que se implementa en el estado de Ceará. En Brasil, 69% de los sujetos diagnosticados con TB se hicieron pruebas para VIH, con 10% de porcentaje de coinfección en 2014. En Ceará, los exámenes están debajo de la media nacional con 58,2% y el porcentaje de coinfección es de 14,6%, con tendencia de crecimiento en los últimos dos años (5, 11).

En el sistema de salud brasileño, los SAE son el lugar de preferencia para el manejo de individuos coinfectados por TB/VIH (9). El desafío para los administradores de salud de Ceará es promover la ampliación del acceso a los cuidados recomendados en el Protocolo de coinfección por TB/VIH mediante la inversión en el pleno funcionamiento de la red de atención estatal y los municipios (6).

En el caso de la coinfección por TB/VIH, se postula que existen barreras relacionadas a la resistencia de los equipos profesionales para asumir la responsabilidad sanitaria por los coinfectados por TB/VIH, así como acciones de monitoreo realizadas de un modo fragmentario por los equipos de administración locales y regionales de los programas de control de sida y del programa de control de tuberculosis.

En base a los problemas señalados, este estudio pretende identificar estrategias de mejora en la adopción del protocolo de manejo TB/VIH mediante investigación evaluativa, con la participación de la red de servicios, administrados y profesionales como agentes clave de investigación y con el compromiso de mejora de acceso a los usuarios.

## MATERIALES Y MÉTODOS

### Diseño del estudio

**FIGURA 1.**
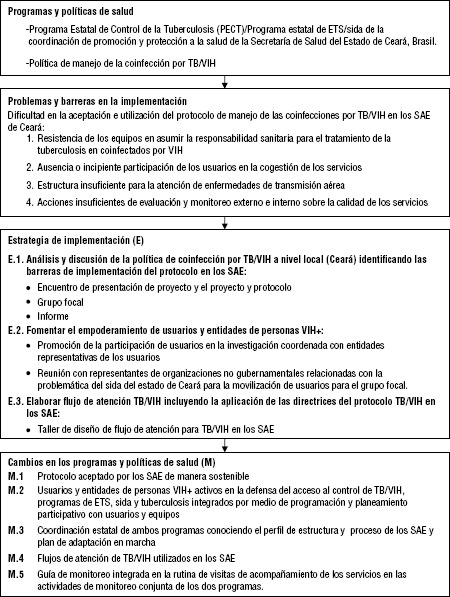
Flujo para la implementación del manejo de la coinfección por TB/VIH en los servicios de asistencia especilizada (SAE) en Ceará, Brasil, 2015.

ETS, enfermedades de transmisión sexual; SIDA, síndrome de inmunodeficiencia adquirido; VIH, vírus dela inmunodeficiencia humana.

Este proyecto se desarrolló siguiendo la metodología iPIER, una nueva iniciativa que tiene el propósito de apoyar mejoras en la ejecución de programas a través de investigaciones integradas en ellos acerca de su ejecución, desarrollado por la Alianza para la Investigación en Políticas y Sistemas de Salud (AHPSR), en colaboración con la Organización Panamericana de la Salud (OPS). El modelo iPIER jerarquiza a los ejecutores de programas como agentes clave de investigación con el objetivo de entender las fallas en los sistemas de salud que crean barreras a la implementación, también permite identificar las soluciones a estas barreras. La investigación sobre la ejecución de programas integrada en los procesos existentes apoya su efectividad y políticas de salud eficaces a través de la utilización de la investigación que se llevó a cabo como parte del proceso de implementación. Una descripción detallada de la aplicación de la metodología de investigación se incluye en el documento conceptual iPIER (12) y en el estudio sobre evaluación de la implementación del protocolo en el tratamiento de TB-VIH-sida en los servicios de asistencia especializados (SAE) en el estado de Ceará (6). Para realizar la evaluación, el equipo elaboró un diagrama de flujo que resume las etapas del estudio (figura 1).

El estudio involucró a dos instituciones públicas de salud y una organización no gubernamental, la Red Nacional de Personas Viviendo con VIH. El equipo de estudio fue formado por cinco investigadores: tres del programa de tuberculosis, uno del programa de VIH-sida y un investigador científico.

El protocolo de investigación fue sometido a los comités de la plataforma Brasil en febrero de 2105, con registro CAAE 42719815.3.000.5469, Resolución 466/2012 del Consejo Nacional de Salud. Después de la aprobación en el país, fue enviado al comité de Ética de la Organización Panamericana de la Salud (PAHOERC 2015.04-0021) y se obtuvo la aprobación final de ambos comités en abril de 2015.

### Lugar del estudio

El estado de Ceará está ubicado en la región Nordeste de Brasil, tiene un territorio de 148 825 km2 y una población de 8 452 381 habitantes. El 75% de la población está concentrada en áreas urbanas, debido a factores climáticos, falta de agua y pobreza en el campo. Las vulnerabilidades individuales y sociales contribuyen con la tendencia de diseminación de la tuberculosis y la epidemia de sida (11, 13).

### Recolección de información

La recolección de la información se realizó entre abril y junio de 2015, con base en fuentes de datos secundarios: los informes del Catastro Nacional de Establecimientos de Salud (CNES), el Sistema de Información de Agravios de Notificación (SINAN) y el Sistema de Información de Mortalidad (SIM). Se trazó la línea de base de estructura y procesos de los 22 servicios por medio del llenado de una guía con siete bloques de preguntas sobre: capacidad instalada y estructura física, recursos humanos y materiales, acciones de investigación de casos con diagnóstico de tuberculosis, vigilancia e indicadores, medios diagnósticos, e intervención y terapias. En la etapa cualitativa se buscó, a través de los grupos focales, conocer la experiencia de acceso, utilización y posibles barreras que podrían influir en la implementación del protocolo, con administradores de servicios, profesionales y usuarios.

Las principales fuentes de datos primarios fueron los grupos focales. Las herramientas de recolección de datos fueron: guía de temas conductores sobre la opinión acerca de los servicios ofrecidos para grupo focal ajustado por las características de los participantes y del temario conductor (para usuarios: tuberculosis, acceso y opinión sobre el servicio, y para profesionales y administradores: conocimiento de la existencia del protocolo, disponibilidad en el servicio, utilización, aceptación e implementación).

### Análisis de datos

Se organizaron los datos cuantitativos de nivel de complejidad del sistema de salud, infraestructura para exámenes, in-sumos farmacéuticos, investigación de contactos, tratamientos de ILTB en un banco de datos e introducidos al *software* STATA® 11.0. La sistematización de los datos se expuso a los administradores y profesionales de salud en un seminario. Los datos cualitativos se obtuvieron en los grupos focales, fueron transcritos y sometidos a análisis de temática de contenidos y constituyó “un conjunto de técnicas de análisis de diálogos, que utiliza procedimientos sistemáticos y objetivos de descripción del contenido de los mensajes, cuya intención es la inferencia de conocimientos relativos a las condiciones de producción o recepción” (14).

## RESULTADOS

### Estructura de la red de atención a personas viviendo con VIH/sida en el estado de Ceará

**FIGURA 2.**
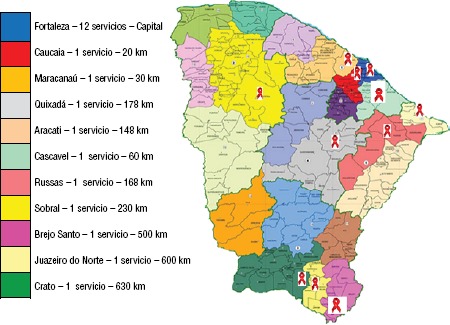
Localización de los servicios de atención especializada (SAE) y su distancia de la capital: Fortaleza, Ceará, Brasil, 2015.

La red de atención a personas con infección por VIH en el estado de Ceará está formada por 22 servicios (SAE), 12 en la capital y 10 en el interior, donde la epidemia tiene mayor relevancia epidemiológica (figura 2).

**FIGURA 3.**
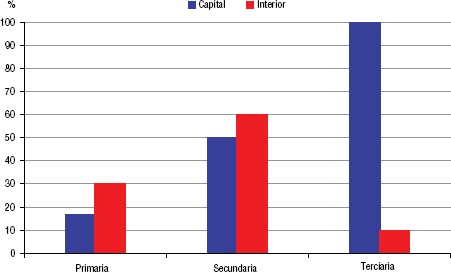
Perfil de inserción de los SAE por nivel de complejidad del sistema de salud, disponibilidad de área física y sustentabilidad de los servicios, Ceará, 2015.

La distribución de los SAE en la red estatal de salud varió en relación a su nivel de inserción en el Sistema Único de Salud (SUS, atención primaria, secundaria y terciaria), esto contribuyó para definir el perfil de estructura, la disponibilidad de área física y los equipos (figura 3).

**FIGURA 4.**
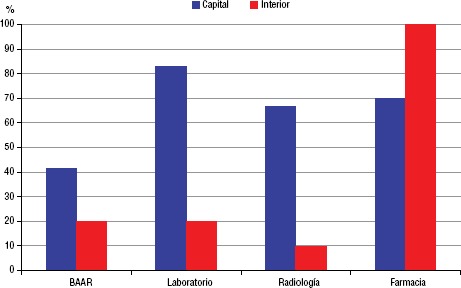
Infraestructura de los servicios de asistencia especializada (SAE) para hacer frente a la coinfección por TB/VIH en el estado de Ceará, Brasil, 2015.

TB, tuberculosis; HIV, virus de inmunodeficiencia humana; BAAR, bacilo ácido-alcohol resistente.

Se observó que 10% de los servicios realizaban exámenes de radiología y 20% hacían examen de esputo para detección del bacilo ácido-alcohol resistente (BAAR) para pacientes con VIH. Los servicios del interior del estado, en comparación con los de la capital, tienen deficiencias en la infraestructura de equipamientos y en la capacitación de los equipos, aunque la estructura física de la mitad de ellos sea superior a los servicios de la capital (figura 4).

Al estudiar los equipos de los 22 SAE, se observó que apenas seis servicios (27,2%) tenían una infraestructura compleja con equipos multidisciplinarios, laboratorio, radiología, farmacia, equipamientos y área física (figura 4). El resto de los servicios estaban compuestos solo por médicos y enfermeras. La mayoría de los profesionales eran médicos (72), seguidos de enfermeras (40), farmacéuticos (23), asistentes sociales (22), psicólogos (22) y bioquímicos (16). En relación a la capacitación de los profesionales en el manejo de la coinfección TB/VIH, se encontró que, en el período 2012–2014, los equipos médicos de 14 servicios (63,6%) y los equipos de enfermería de 11 (50%) habían recibido entrenamiento, y que los asistentes sociales, bioquímicos y psicólogos, en la mayoría de los servicios, no habían recibido entrenamiento específico hasta 2015.

**FIGURA 5.**
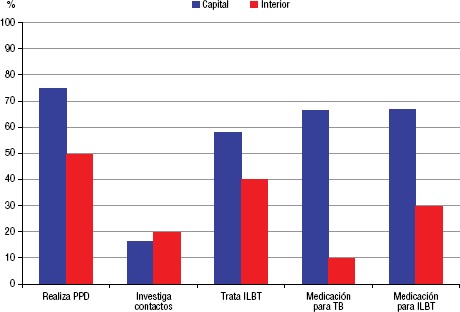
Acciones de manejo de la coinfección por TB/VIH en los servicios de asistencia especializada (SAE) en el estado de Ceará, Brasil, 2015.

TB, tuberculosis; VIH, virus de inmunodeficiencia humana; PPD, prueba cutánea de derivado proteínico purificado; ILBT, infección latente por tuberculosis.

Los servicios de la capital mostraron mejor organización para el manejo de la coinfección TB/VIH: en la mayoría (75%) se realizaba PPD, se trataban ILTB y se disponía de esquemas de tratamiento para TB e ILTB. La mitad de los servicios del interior ofrecían la PPD, 10% disponían de fármacos específicos para tuberculosis y 30% tuvieron disponibilidad de tratamiento para ILTB (figura 5).

### Aspectos cualitativos de la implementación del protocolo

El método cualitativo de grupos focales favoreció los discursos y el intercambio de ideas entre los participantes de cada grupo (administradores y gestores de servicios, profesionales y usuarios).

En relación a la atención, la población usuaria de los SAE refiere:

“He presenciado, escuchado, historias, ¿sí? (...) Tengo miedo de ir al puesto [unidad de atención primaria para tratar tuberculosis] y que alguien sepa que soy VIH y vaya a contarle a mi familia”.

“Pero sí, las unidades necesitan recibir habilidades para tratar tuberculosos… si la persona sabe que uno tiene tuberculosis ya se queda a una cierta distancia, abriendo las puertas…”.

“[La tuberculosis] es una enfermedad que todavía tiene mucho más estigma que el propio VIH”.

Cómo recibieron el protocolo TB/VIH los equipos:

“Solo las enfermeras recibieron el protocolo”. (Administrador)

“Yo no conozco el protocolo (…) el médico está siempre pidiendo el examen de esputo, que se hace ahora, un test rápido, ¡sí! Pero la cuestión es que yo no tengo una buena base del examen”. (Administrador)

“Si no funciona es porque son ‘difíciles’. Puedes tener todo el equipo…. Todo con protocolo se puede tener todo para funcionar, pero si la persona no quiere, la cosa no va”. (Administrador)

Puntos a favor para la ejecución del protocolo TB/VIH:

“No tengo mucho que reclamar. Es bien práctico para leer”. (Profesional)

“Yo creo que cuando el equipo es bueno y tú lees, tú consigues llevarlo a cabo”. (Profesional)

“Cada quien tiene un protocolo en el servicio”. (Profesional)

“La verdad, yo hice el entrenamiento de TB, volví para hacer el servicio y recibí el protocolo TB-VIH”. (Profesional)

“… estoy hablando de cosas de VIH-TB, yo nunca hice ningún entrenamiento, me mandaron allá a ese lugar, ¿entendió? Y todo lo que aprendí fue leyendo en internet y viendo allá lo que sucedía”. (Profesional)

Implementación del protocolo en la práctica:

“La tuberculosis dentro del servicio [SAE] ahora es novedad; antes, nuestros pacientes de VIH, cuando se diagnosticaban, iban a tratarse a la unidad básica de salud [atención primaria]. Y ahora este nuevo sistema [Protocolo] (…) cuando se abrió, fue que se comenzó con el tratamiento dentro de nuestra unidad”. (Administrador)

En los grupos focales, las barreras de implementación identificadas incluyen (aunque no están limitadas a esto): discriminación y estigmatización con la tuberculosis, recursos humanos insuficientes, bajo compromiso para atender las dos enfermedades, diferencias en las recomendaciones y frecuencia de las consultas para TB/VIH, sobredemanda en los servicios especialistas e inadecuada estructura para la atención de las enfermedades transmisibles en los servicios.

## DISCUSIÓN

En relación al contexto de los servicios, se encontró que el modelo de organización, en su mayoría, está dirigido a la atención a las personas con VIH/SIDA y en solo seis (27%) de los servicios se llevan a cabo acciones de control específicas para la tuberculosis. Allí donde existen, se realizan de forma desarticulada, con modificaciones de las recomendaciones del protocolo de manejo de coinfección, incluida la infección tuberculosa latente. Se identificó una deficiencia en la capacitación profesional para atender la demanda de personas coinfectadas por TB/VIH. Esto sugiere una baja inversión por parte del Ministerio y del Estado en la actualización permanente necesaria para todo el equipo de atención.

Los servicios no tienen estructura física compleja y adecuada para la atención de las personas coinfectadas de TB/VIH en términos de bioseguridad, acceso a fármacos y exámenes complementarios de diagnóstico de TB (figura 4) y consultas con especialistas. De esta manera, se dificulta el diagnóstico oportuno de la tuberculosis.

Entre las estrategias de implementación, se ofreció una retroalimentación referente al perfil de la infraestructura. En esa ocasión, se discutieron las medidas administrativas de adaptación del espacio físico y la redefinición de flujos internos, para hacer posible la atención de personas coinfectadas con TB/VIH en los servicios especializados. Los administradores, según los resultados del estudio, estarán en condiciones de producir material adecuado para la mayor difusión de las normas para equipos y usuarios ante la imposibilidad de cambios inmediatos en la estructura física de los servicios. Se estableció el monitoreo integrado con representantes de los programas de tuberculosis y VIH/sida para promover la aceptación, adopción y utilización del protocolo como estrategia de ampliación de la oferta y cobertura de atención a los coinfectados por TB/VIH.

Para monitorear la adopción del protocolo, se usaron indicadores como el número de notificaciones de casos de nuevos de coinfección por TB/VIH realizado por los equipos de los SAE en el SINAN, la demanda de esquemas terapéuticos para tuberculosis para usuarios de los SAE, el interés de los administradores y profesionales en la demanda y oferta de capacitación, la solicitud de insumos para exámenes de diagnóstico de TB e inserción del tema TB/VIH en las agendas de las instancias colegiadas del SUS.

El protocolo de manejo de la tuberculosis exige adhesión al tratamiento, con tomas diarias y supervisadas de la medicación, un tipo de actividad que es adecuada para el ámbito de la atención primaria, y que los servicios especialistas (SAE) no pueden realizar por falta de infraestructura y por la insuficiente comprensión, por parte del equipo, de que esta es una función de atención primaria (1, 3, 8, 15). Al analizar el problema desde la visión del paciente, se detectan discursos que denotan que el usuario preferiría ser tratado en el ámbito de la atención primaria, por cercanía a su domicilio, ya que de otra manera el traslado diario para realizar el tratamiento supervisado es difícil (9, 10). Por otro lado, hay un grupo de pacientes que teme la revelación social de su estado serológico (estigma social) y preferiría realizar el tratamiento de forma autónoma, sin necesidad de supervisión profesional. Las dos situaciones sugieren que sería necesaria la articulación para el respeto al derecho de decisión de los pacientes (13). En la realidad del sistema de salud, las normas y directrices deben adecuarse a las necesidades del usuario y su autonomía, respetando los principios de descentralización y las posibilidades locales.

Es probable que este hecho sea resultado del modelo de atención primaria existente y, por lo tanto, resulta lógico que los recursos para aumentar la capacidad sean destinados para profesionales que actúan en la primera línea de la atención primaria. Otro factor crítico que dificulta la ejecución del protocolo TB/VIH es la escasa disponibilidad de insumos y fármacos para el tratamiento de la tuberculosis en los servicios especializados: solo ocho de esos servicios disponían de los esquemas antibióticos apropiados para la tuberculosis.

Existe un factor de tipo sistémico relacionado al flujo de distribución de insumos y fármacos para la tuberculosis, por el cual son entregados con prioridad para la red básica. Las coordinaciones de asistencia farmacéutica de las regionales de salud, responsables de abastecer la red de salud, no siempre son sensibles a la necesidad de modificar la distribución. Tal situación sugiere un cambio en el diálogo con la asistencia farmacéutica, que es una de las estrategias de implementación a ser adoptadas por las administraciones de las áreas técnicas de TB y sida para la implementación del protocolo; es decir, establecer articulación con los representantes regionales de asistencia farmacéutica para redefinir flujos de entrega de tratamientos en los SAE.

Para la promoción de los cambios establecidos en pacto con los equipos, se realizaron visitas de monitores integradas entre los dos programas (TB y VIH), con el objetivo de acompañar la ejecución de los nuevos flujogramas de atención y orientarlos cuando fuera necesario. Se incorporaron cambios en los procesos en el instrumento de monitoreo de los servicios, con la intención de planificar la educación permanente y hacer pactos con los administradores municipales y regionales de salud para monitoreo de los indicadores.

Los problemas identificados en la presente investigación son útiles en los procesos de aprendizaje y educación continuos de los equipos y los administradores (12, 15). Las intervenciones de campo en los servicios y junto a los usuarios produjeron algunos indicios de cambios verificados en las entrevistas, las visitas de campo y en el instrumento de autoevaluación respondido en setiembre de 2015 y en el último seminario. Entre ellos, se destacan el aumento de la difusión del protocolo entre administradores, profesionales y representantes de usuarios; la inclusión del protocolo en la pauta de las reuniones de rutina de los servicios; el mayor número de discusiones internas en cuanto a los problemas y las rutas para la incorporación del protocolo en los servicios; y cambios de actitud de los administradores con relación a la implementación percibida por medio de iniciativas, como solicitud de libros de registros para atención de la coinfección, de copias del protocolo, de lugares en el perfeccionamiento con respecto a la tuberculosis y el aumento de la demanda de insumos en la coordinación de tuberculosis por parte de los servicios.

### Conclusión

El modelo iPIER de evaluación posibilitó el contacto directo entre los administradores, ejecutores e investigadores y el compromiso inmediato de un número significativo de personas con la implementación del protocolo. Además, mejoró el conocimiento de las barreras para implementación del protocolo en lo referente a medidas de seguridad y diagnóstico sobre tuberculosis y necesidad de información entre usuarios. Los cambios acontecieron durante el proceso de evaluación, con beneficios en la toma de decisiones. El conocimiento basado en la evidencia científica del protocolo fue trasladado a la práctica de los servicios y adoptado por al menos el 55% de ellos.

### Agradecimientos

Los autores agradecen el apoyo de Ludovic Reveiz, Nhan Tran, Ettiene Langlois, Janaina Sallas, Yurani Sandoval, Sebastián García Martí, Ariel Bardach y todo el equipo del proyecto iPIER.

### Financiamiento

Este trabajo fue financiado por la Alianza para la Investigación en Políticas y Sistemas de Salud (AHPSR), de la Organización Mundial de la Salud (OMS). La Organización Panamericana de la Salud (OPS) brindó cooperación técnica para el desarrollo de este proyecto. En el contexto del programa iPIER, el Instituto de Efectividad Clínica y Sanitaria (IECS) brindó asistencia técnica para el desarrollo del protocolo y la ejecución del proyecto.

### Declaración

Las opiniones expresadas en este manuscrito son responsabilidad del autor y no reflejan necesariamente los criterios ni la política de la *RPSP/PAJPH* y/o de la OPS.
